# Supplementary material for: Classification of Polish Natural Bee Honeys Based on Their Chemical Composition
Source: Molecules. 2022 Jul 28;27(15):4844. doi: 10.3390/molecules27154844 (PMC9369904; doi:10.3390/molecules27154844)
Supplement: Supplementary file 1 [file molecules-27-04844-s001.zip › molecules-1817056-supplementary.pdf]

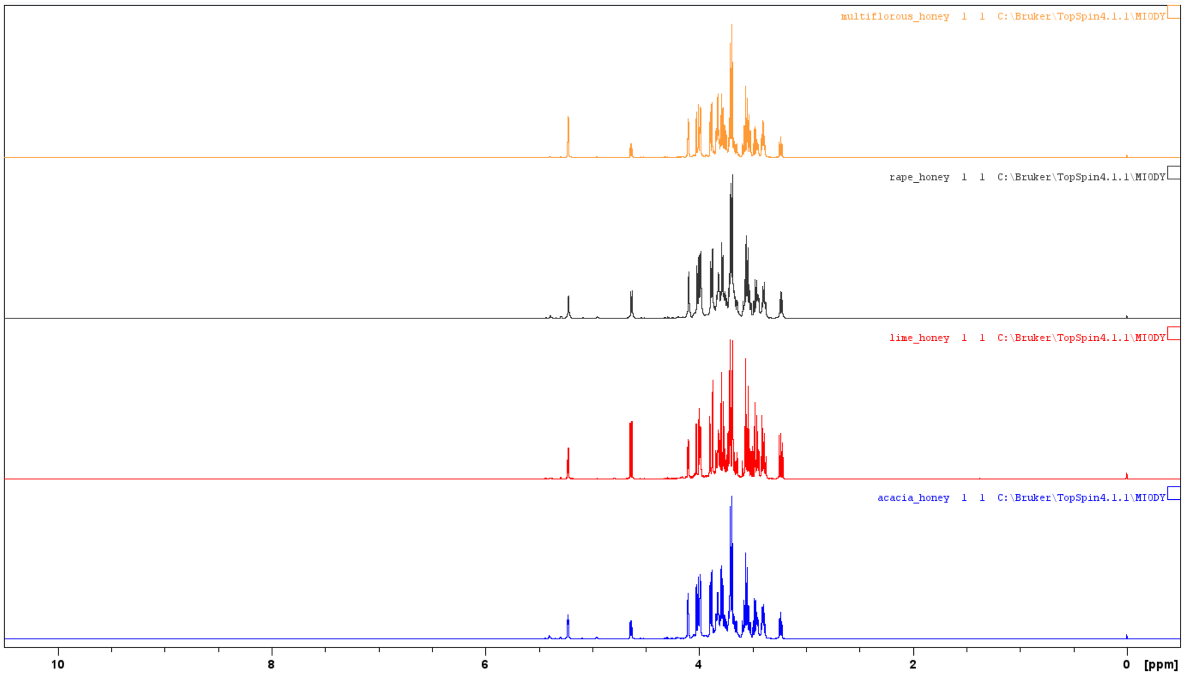

Figure S1. ^{1}H NMR spectra of lime, rape, acacia and multifloral Polish honey samples in spectral region from 0 to 11 ppm.

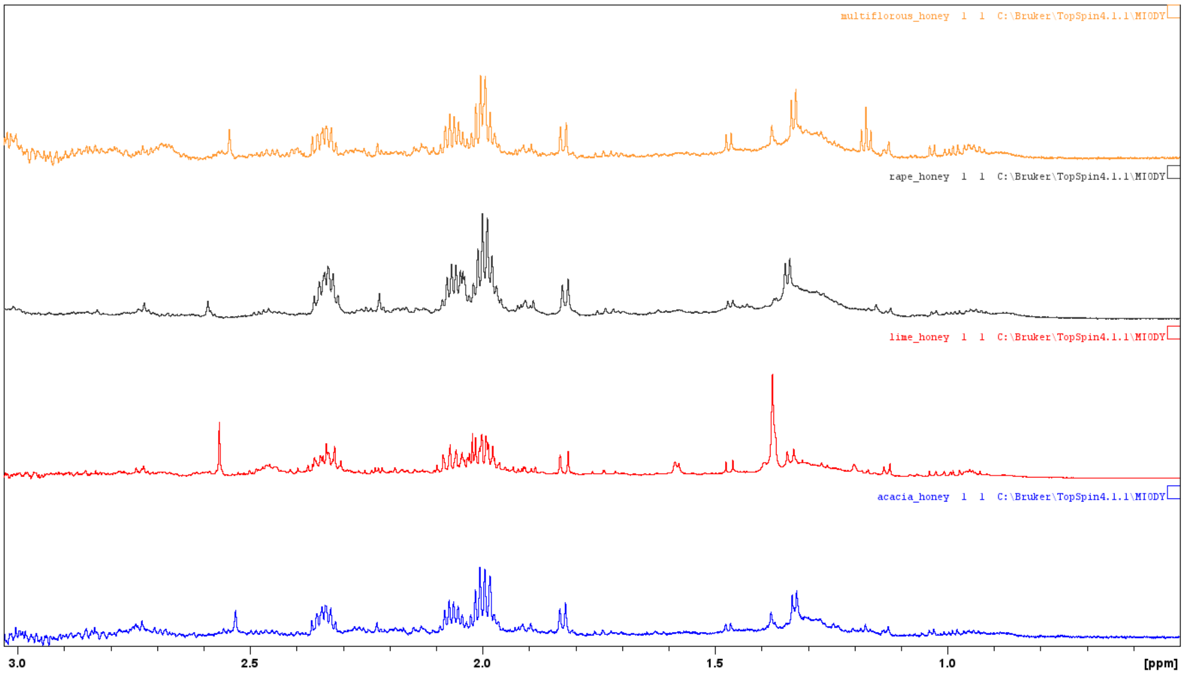

Figure S2. ^{1}H NMR spectra of lime, rape, acacia and multifloral Polish honey samples in spectral region from 0 to 3 ppm.

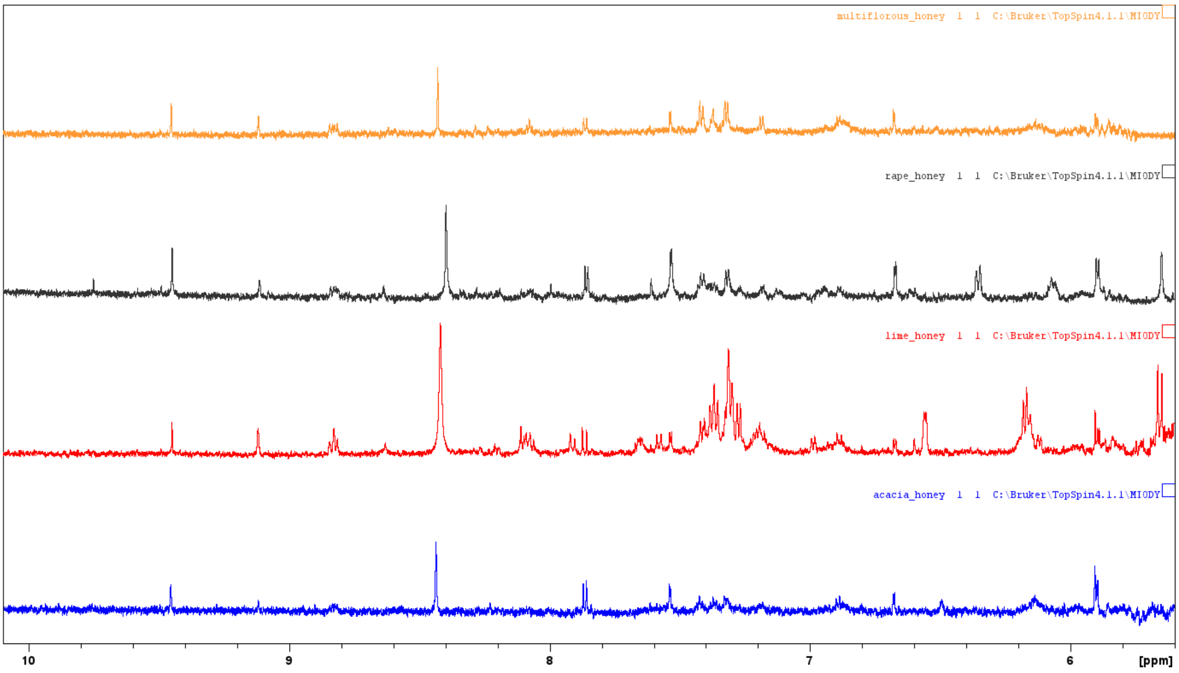

Figure S3. ^{1}H NMR spectra of lime, rape, acacia and multifloral Polish honey samples in spectral region from 5.6 to 10 ppm.

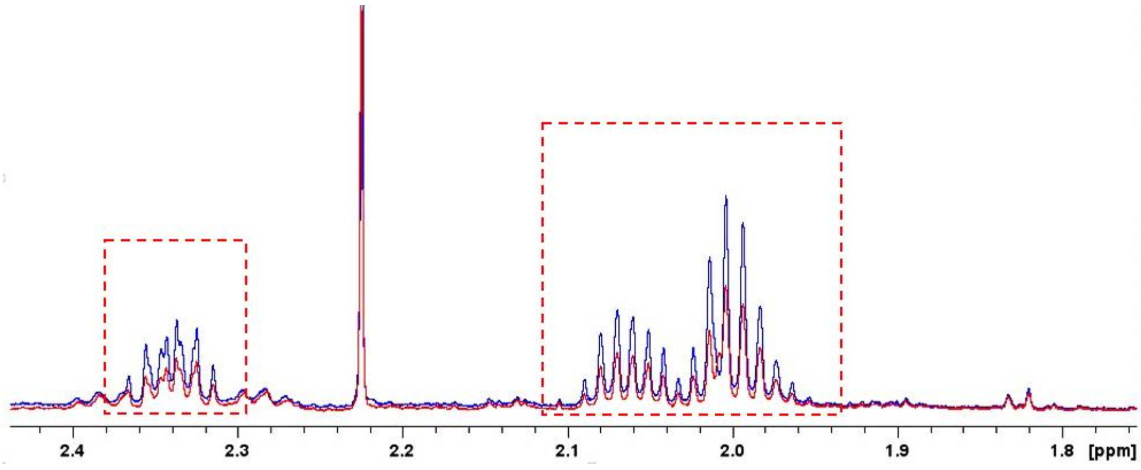

Figure S4. Expanded region of ^{1}H NMR spectrum of accacia honey sample (red spectrum) spiked with proline solution (blue spectrum).

^{13}C NMR spectra of analyzed honeys consisted mainly of fructose (fructofuranose and fructopyranose), glucose and sucrose signals.

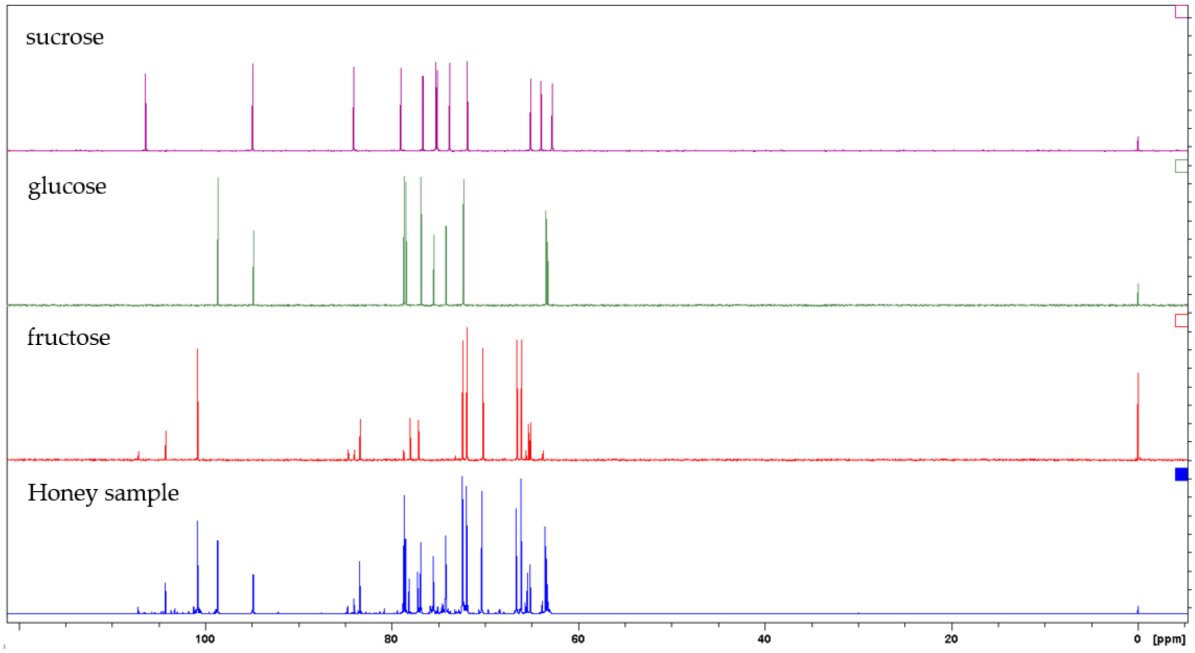

Figure S5. ^{13}C NMR spectrum of lime honey sample with ^{13}C spectra of standard sample of fructose, glucose and sucrose.

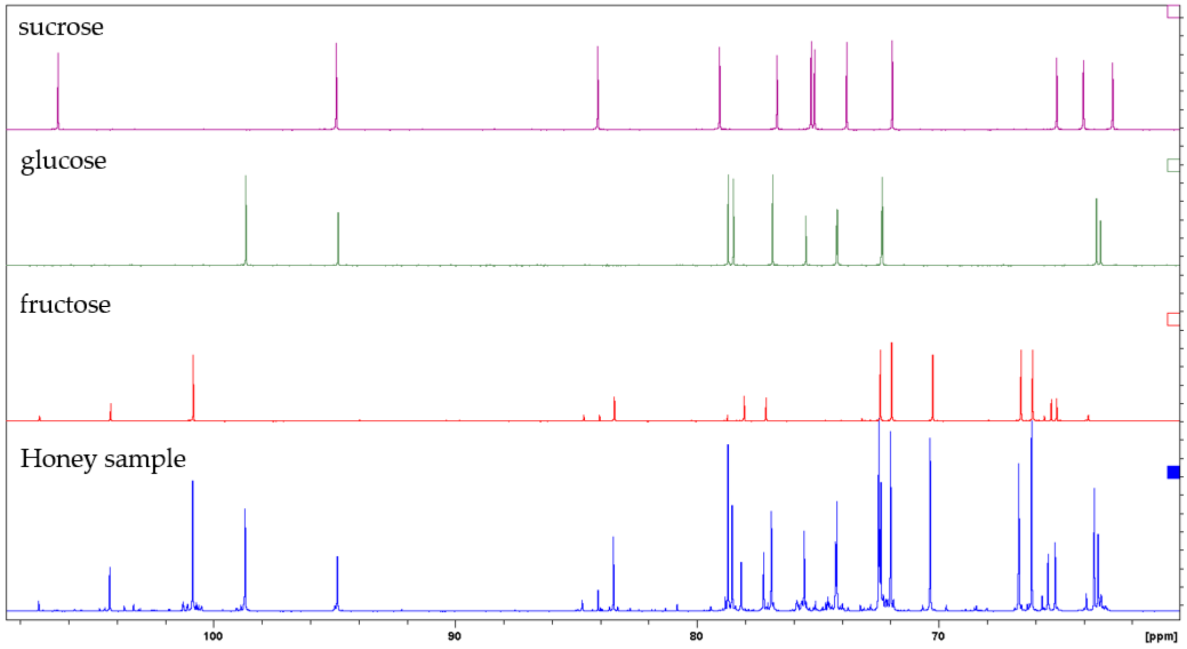

Figure S6. Expanded region of ^{13}C NMR spectrum of lime honey sample with ^{13}C spectra of standard sample of fructose, glucose and sucrose.

Table S1. The main compounds assigned in ^{1}H NMR spectra of honey samples, their diagnostic ^{1}H signals, chemical shifts δ_H, coupling constants, and multiplicities.

	Compound	Chemical shift [ppm] (multiplet)
**1**	Valine	0.97 (d), 1.03 (d), J=7.0 Hz
**2**	Ethanol	1.18 (t), J=7.1 Hz
**3**	Lactic acid	1.33 (d), J=6.9 Hz
**4**	Dimethyl succinic acid	1.37 (s), 2.56 (s)
**5**	Alanine	1.47 (d), J=7.2 Hz
**6**	Proline	1.95-2.10 (m)<br>2.29-2.38 (m)
**7**	Fructose	4.11(d), J=3.7 Hz
**8**	Arabinose	4.49 (d), J=7.8 Hz
**9**	β-glucose<br>α-glucose	4.64 (d), J=7.9 Hz<br>5.22 (d), J=3.8 Hz
**10**	Sucrose	5.44 (d), J=3.8 Hz
**11**	Tyrosine	6.91 (d), J=8.3 Hz<br>7.18 (d), J=8.3 Hz
**12**	Phenylalanine	7.28 (d) J=7.6 Hz<br>7.37 (t)<br>7.40(t)
**13**	5-(hydroxymethyl)furfural	6.67 (d), J=3.7 Hz<br>7.53 (d), J=3.7 Hz<br>9.45 (s)
**14**	4-chlorobenzoic acid	7.58 (d), J=8.5 Hz<br>7.86 (d), j=8.5 Hz
**15**	Formic acid*	8.45 (s)

* Formic acid was not detected in most of the samples. In four samples it was found in small amounts of 0.54 -0.96 mg/kg.

3. Calibration curves for quantified compounds.

To check linearity of proposed method ^{1}H NMR spectra for 8 series of selected compounds were recorded. In the case of glucose solutions (0.48, 0.96, 1.92, 4.21, 8.43, 17.50, 33.71 and 66.82 g/100g) ^{1}H NMR spectra were measured with two different different repetition time: T_r = 6s and 8.5s. Calibration curves for Glc were presented in the main manuscript. Each sample was analyzed in triplicate with random order. The criteria of acceptance: the recovery between 98 to 102%.

Table S2. Accuracy of applied method for Glc solutions measured with repetition time T_r =8,5s.

Numer of sample	Theoretical mass [g]	Experimental mass [g]	Recovery [%]
1	66.82	67.05 ± 0.04	100.34
2	33.71	33.68 ± 0.03	99.91
3	17.50	17.48 ± 0.04	99.86
4	8.43	8.46 ± 0.03	100.35
5	4.21	4.24 ± 0.03	100.71
6	1.92	1.95 ± 0.04	101.56
7	0.96	1.07 ± 0.01	111.45
8	0.48	0.49 ± 0.04	102.08

Table S3. Accuracy of applied method for Glc solutions measured with repetition time Tr = 6s.

Number of sample	Theoretical mass [g]	Experimental mass [g]	Recovery [%]
1	66.82	65.71 ± 0.01	98.34
2	33.71	33.90 ± 0.02	100.56
3	17.50	17.51 ± 0.03	100.06
4	8.43	8.40 ± 0.02	99.64
5	4.21	4.19 ± 0.05	99.52
6	1.92	1.91 ± 0.03	99.47
7	0.96	0.96 ± 0.02	100.00
8	0.48	0.47 ± 0.03	97.91

Calibration curve for fructose

In the case of fructose solutions (0.5, 5.22, 15.10, 30.26, 45.62, 60.12, 85.44 and 100 g/100g) ^{1}H NMR spectra were measured with selected repetition time: $T_r = 6s$. Each sample was analyzed in triplicate with random order. The criteria of acceptance: the recovery between 98 to 102%.

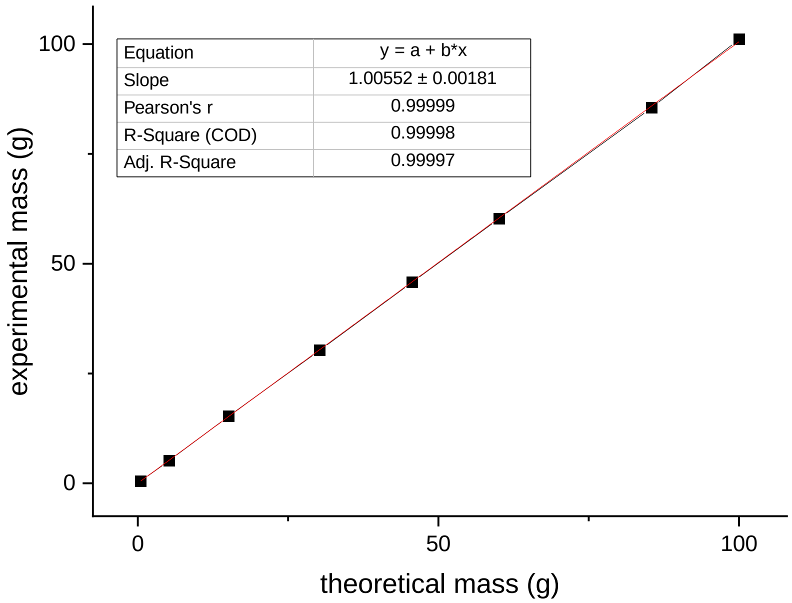

Figure S7. Linearity of the method for fructose with repetition time equals 6s.

Table S4. Accuracy of applied method for fructose solutions measured with repetition time T_r =6 s.

Numer of sample	Theoretical mass [mg]	Experimental mass [mg]	Recovery [%]
1	0.50	0.49± 0.03	98.00
2	5.12	5.17±0.02	100.97
3	15.10	15.24±0.01	100.93
4	30.26	30.31±0.03	101.16
5	45.62	45.78±0.03	100.35
6	60.12	60.29±0.04	100.28
7	85.44	85.47±0.03	100.04
8	100.00	101.1±0.02	101.10

Calibration curve for sucrose

In the case of sucrose solutions (0.05, 0.25, 0.63, 1.44, 2.88, 5.22, 7.89 and 10 g/100g) ^{1}H NMR spectra were measured with selected repetition time: $T_r = 6s$. Each sample was analyzed in triplicate with random order. The criteria of acceptance: the recovery between 98 to 102%.

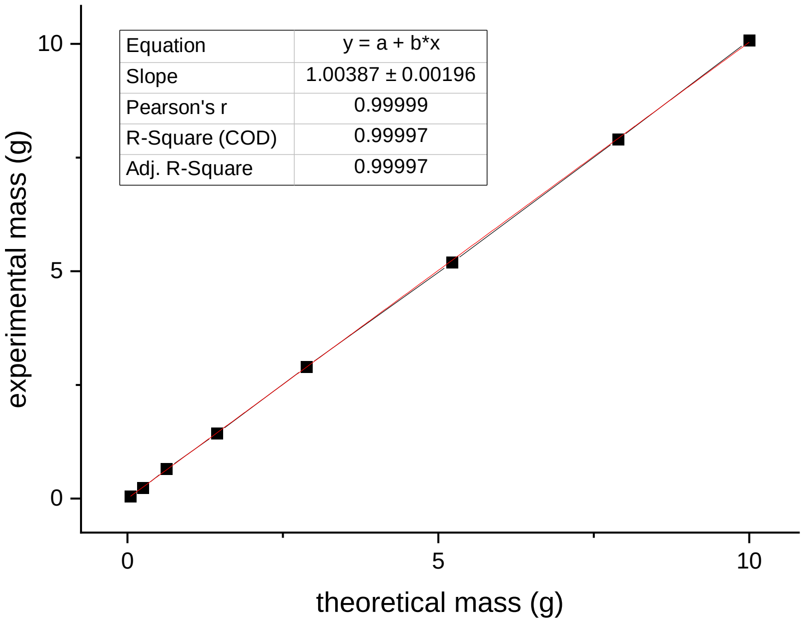

Figure S8. Linearity of the method for sucrose with repetition time equals 6s.

Table S5. Accuracy of applied method for sucrose solutions measured with repetition time T_r =6 s.

Numer of sample	Theoretical mass [mg]	Experimental mass [mg]	Recovery [%]
1	0.05	0.051± 0.01	102.00
2	0.25	0.24±0.03	96.00
3	0.63	0.65±0.01	103.17
4	1.44	1.43±0.02	99.31
5	2.88	2.90±0.03	100.69
6	5.22	5.19±0.02	99.43
7	7.89	7.90±0.02	100.13
8	10.00	10.08±0.01	100.80

Calibration curve for Alanine

In the case of Ala solutions (0.49, 1.11, 1.64, 4.34, 6.03, 7.25, 8.48 and 10 mg/100g) ^{1}H NMR spectra were measured with selected repetition time: $T_r = 6s$. Each sample was analyzed in triplicate with random order. The criteria of acceptance: the recovery between 98 to 102%.

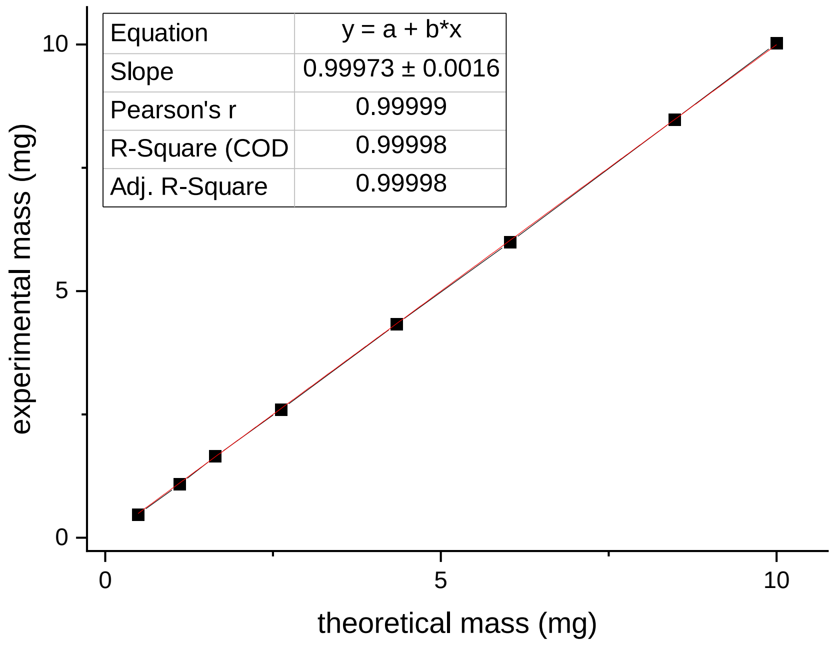

Figure S9. Linearity of the method for Ala with repetition time equals 6s.

Table S6 Accuracy of applied method for Ala solutions measured with repetition time T_r =6 s.

Numer of sample	Theoretical mass [mg]	Experimental mass [mg]	Recovery [%]
1	0.49	0.47±0.01	95.92
2	1.11	1.08±0.02	97.30
3	1.64	1.65±0.01	100.61
4	2.62	2.60±0.01	99.24
5	4.34	4.33±0.02	99.77
6	6.03	5.99±0.02	99.34
7	8.48	8.48±0.02	100.00
8	10.00	10.03±0.02	100.30

Calibration curve for Phenylalanine

In the case of Phe solutions (0.50, 1.52, 3.39, 6.55, 9.40, 11.53, 17.38 and 20 mg/100g) ^{1}H NMR spectra were measured with selected repetition time: $T_r = 6s$. Each sample was analyzed in triplicate with random order. The criteria of acceptance: the recovery between 98 to 102%.

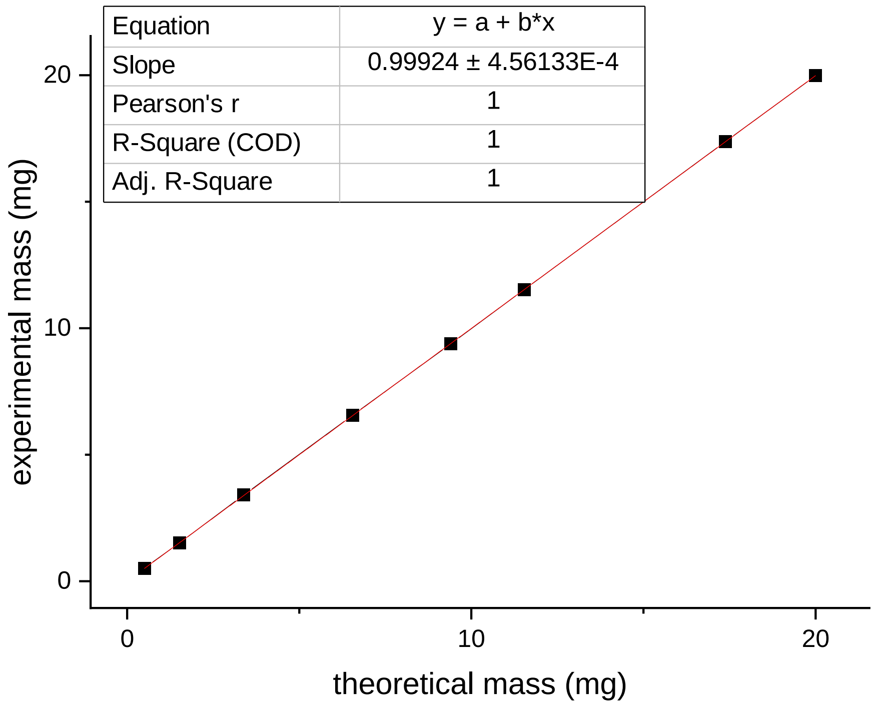

Figure S10. Linearity of the method for Phe with repetition time equals 6s.

Table S7. Accuracy of applied method for Phe solutions measured with repetition time T_r =6 s.

Numer of sample	Theoretical mass [mg]	Experimental mass [mg]	Recovery [%]
1	0.50	0.52± 0.04	104.00
2	1.52	1.51±0.04	99.34
3	3.39	3.41±0.04	100.59
4	6.55	6.56±0.03	100.15
5	9.40	9.38±0.04	99.79
6	11.53	11.52±0.03	99.91
7	17.38	17.37±0.01	99.94
8	20.00	19.98±0.02	99.90

Calibration curve for tyrosine

In the case of Tyr solutions (0.48, 1.55, 3.26, 7.31, 10.45, 13.50, 16.40 and 20.07 mg/100g) ^{1}H NMR spectra were measured with selected repetition time: T_r = 6s. Each sample was analyzed in triplicate with random order. The criteria of acceptance: the recovery between 98 to 102%.

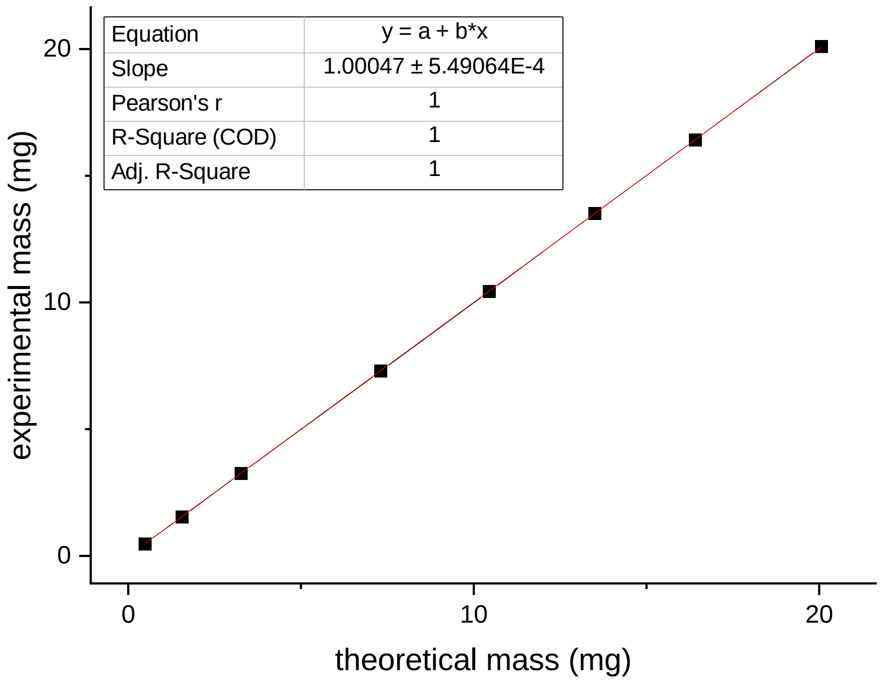

Figure S11. Linearity of the method for Tyr with repetition time equals 6s.

Table S8. Accuracy of applied method for Tyr solutions measured with repetition time T_r =6 s.

Numer of sample	Theoretical mass [mg]	Experimental mass [mg]	Recovery [%]
1	0.48	0.48± 0.02	100.00
2	1.55	1.53±0.03	98.71
3	3.26	3.25±0.02	99.69
4	7.31	7.29±0.03	99.73
5	10.45	10.43±0.03	99.81
6	13.50	13.51±0.01	100.07
7	16.41	16.42±0.02	100.06
8	20.07	20.10±0.01	100.15

Calibration curve for proline

In the case of Pro solutions (0.52, 1.57, 4.98, 10.86, 22.18, 49.87, 67.32 and 80.12 mg/100g) ^{1}H NMR spectra were measured with selected repetition time: $T_r = 6s$. Each sample was analyzed in triplicate with random order. The criteria of acceptance: the recovery between 98 to 102%.

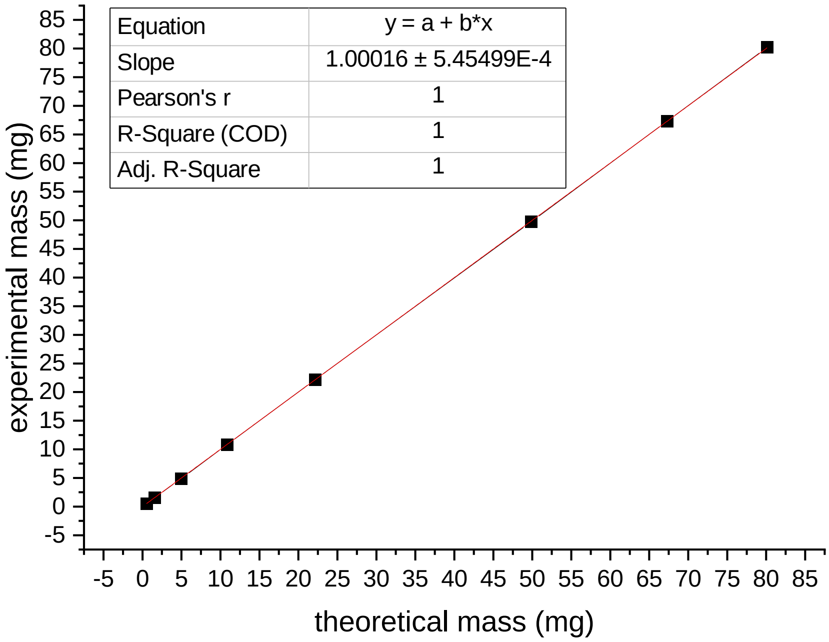

Figure S12. Linearity of the method for Pro with repetition time equals 6s.

Table S9. Accuracy of applied method for Pro solutions measured with repetition time T_r =6 s.

Numer of sample	Theoretical mass [mg]	Experimental mass [mg]	Recovery [%]
1	0.52	0.54± 0.03	103.85
2	1.57	1.59±0.01	98.59
3	4.98	4.91±0.02	99.82
4	10.86	10.84±0.03	100.09
5	22.18	22.20±0.03	99.76
6	49.87	49.75±0.03	100.04
7	67.32	67.35±0.04	100.10
8	80.12	80.20±0.02	101.27

Calibration curve for hydroxymethylfurfural

In the case of HMF solutions (0.50, 0.97, 1.48, 2.51, 5.52, 10.11, 18.66 and 30.25 mg/1000g) ^{1}H NMR spectra were measured with selected repetition time: $T_r = 6$s. Each sample was analyzed in triplicate with random order. The criteria of acceptance: the recovery between 95 to 105%.

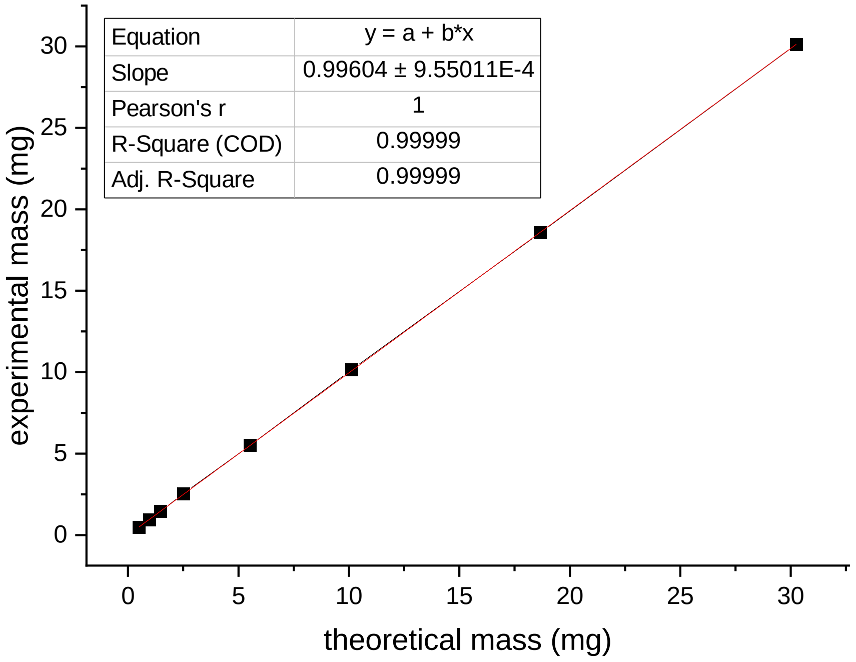

Figure S13. Linearity of the method for HMF with repetition time equals 6s.

Table S10. Accuracy of applied method for HMF solutions measured with repetition time T_r =6 s.

Numer of sample	Theoretical mass [mg]	Experimental mass [mg]	Recovery [%]
1	0.50	0.48± 0.04	96.00
2	0.97	0.95±0.03	97.94
3	1.48	1.46±0.03	98.65
4	2.51	2.55±0.02	101.59
5	5.52	5.50±0.03	99.64
6	10.11	10.14±0.02	100.30
7	18.66	18.56±0.01	99.46
8	30.25	30.12±0.01	99.57
